# Determination of aflatoxin M1 in breast milk and related factors

**DOI:** 10.1590/1806-9282.20211077

**Published:** 2022-08-19

**Authors:** Reyhan Aydın Doğan, Merve Afacan, Mehmet Ozdemir

**Affiliations:** 1 Karabuk University, Faculty of Health Sciences, Department of Midwifery – Karabük, Turkey.; 2 Karabuk University, Faculty of Medicine, Department of Medical Pharmacology – Karabük, Turkey.

**Keywords:** Aflatoxin M1, Human milk, ELISA, Infant

## Abstract

**OBJECTIVE:**

Breastfeeding in women with aflatoxin M1 exposure may be a risk factor for the newborn. Thus, it is crucial to determine aflatoxin M1 levels in breast milk and raise mothers’ awareness about nutrition in lactation and other periods. This study was carried out to determine aflatoxin M1 contamination in milk samples taken from mothers who gave birth.

**METHODS:**

The study was carried out in the postpartum department of Training and Research Hospital between December 31, 2018, and June 31, 2019, and 90 breastfeeding mothers were included in the study.

**RESULTS:**

A total of 75 (83.3%) of the examined samples were found positive. The mean aflatoxin M1 ratio in positive samples was 12.16 pg/mL (5.00–23.18 pg/mL). Mothers’ consumption of processed food was associated with aflatoxin M1 levels (p=0.043). It was determined that the aflatoxin M1 levels of mothers who consumed processed food products 1 or 2 times a month were 3.22 times lower than those who consumed 1–2 times a week.

**CONCLUSIONS:**

This study emphasized the importance of monitoring aflatoxin M1 levels in breast milk for infant health. It is thought that nutrition education given to mothers during pregnancy will significantly impact aflatoxin M1 results. In addition, the dangers of mycotoxins in mother-infant nutrition should be emphasized regularly in health education.

## INTRODUCTION

Breast milk alone contains all the nutrients necessary for optimal growth and development of infants for the first 6 months^1^. Depending on the mother’s nutritional style, the milk contains nutritive and immunologically beneficial components as well as the contaminants it is exposed to. Thus, it is transferred to the baby in contaminants along with the beneficial components. One of these contaminants is mycotoxins, which occur naturally under appropriate heat and humidity and produce various toxic metabolites by fungi^2,3^.

Among the aflatoxins, the most potent hepatocarcinogen and hepatotoxic is AFB_1_^4^. This aflatoxin is produced by the fungus *Aspergillus flavus*. Lactation women and animals consuming food contaminated with AFB_1_ undergo hydroxylation by the AFB_1_ cytochrome P450 enzyme system. AFB_1_ is known to transform into its primary metabolite, aflatoxin M1 (AFM_1_), which is 10 times less carcinogenic than itself in 12–24 h. It is also accepted that AFM_1_ is excreted from the body with urine and breast milk^4^. The hydroxylated metabolite of aflatoxins, AFM_1_, is observed in human breast milk^5^. Considering the importance of breastfeeding in the development of newborn infants, the presence of mycotoxins and other contaminants in breast milk should be continuously evaluated. Exposure to aflatoxin can have devastating consequences, especially in newborns whose immune system has not yet developed. In this respect, infants who consume contaminated breast milk containing AFM_1_ are at risk of developing hepatocellular carcinoma, stunted development, encephalopathy, and visceral fatty degeneration^5,6^. Breast milk, which is essential for the growth and development of newborns’ immune systems, should be as far away from these mycotoxins as possible^7^.

Aflatoxin M1 (AFM_1_) concentrations in breast milk were measured in Colombia^8^, Australia^9^, the United Arab Emirates (UAE)^10^, Egypt^11^, Turkey^2–4^, Iran^12^, Brazil^13^, Italy^14^, and Norway^15^. AFM_1_ has been detected in human breast milk at various concentrations in various research^5^. Sierra Leone (0.80 ng/L) and the UAE had the lowest and highest quantities of AFM_1_ in human breast milk, respectively. The Gambia, Tanzania and Jordan had the lowest prevalence of AFM_1_ in human breast milk (2%), whereas the Gambia, Tanzania, and Jordan had the highest (100%). The continents of America (10.30 ng/L) and South-East Asia (358.99 ng/L) have the lowest and highest quantities of AFM_1_ in human breast milk, respectively. In addition, the West Pacific (7%) and Africa (52%) continents have the lowest and highest incidence of AFM_1_ in human breast milk, respectively^5^.

Breastfeeding has been shown to be a risk factor for babies in women who have been exposed to AFM_1_^5^. Thus, it is crucial to determine AFM_1_ levels in breast milk and raise mothers’ awareness about nutrition in lactation and other periods. In addition, it is essential to reduce the toxic effects of AFM_1_ by providing information on protection from AFM_1_ exposure. In the literature review, few studies investigating AFM_1_ exposure in breast milk were found in Turkey^2–4^. When these studies were examined, no study was found in the Black Sea region. The Black Sea region is a geographical region that receives constant precipitation. It has been reported that the prevalence of AFM_1_ in breast milk increases significantly with increasing annual mean precipitation^5^. Considering the regional variation of AFM_1_ exposure, investigating the presence of AFM_1_ in breast milk in the Black Sea, which is a region that receives continuous rainfall, will contribute to the literature. The aims of this study were to measure the levels of AFM_1_ in the breast milk of mothers who had given birth in the area of Black sea and to identify demographic and dietary factors associated with these levels.

## METHODS

### Study design

This is a toxicological analysis laboratory study. Permission was obtained from the Human Research Ethics Committee (dated May 30, 2018 and no. 6/15) and the institute where the research was conducted.

### Participants

Milk samples were collected between December 31, 2018, and June 31, 2019. Data were collected by face-to-face interview technique. A questionnaire consisting of 22 questions was used to collect milk samples, including sociodemographic information about the mothers, nutritional habits, living environment characteristics, and 24-h food consumption information. A total of 90 lactating women who gave birth were included in the present study to analyze AFM_1_ levels in breast milk.

### Data analysis

#### Aflatoxin M1 analysis in breast milk samples

Aflatoxin M1 (AFM_1_) levels were determined using competitive ELISA and AFM_1_ Ridascreen (R1121) commercial assay kits. The lowest detection limit of the AFM_1_ Ridascreen commercial test kit is 5 ppt for milk samples. Hence, breast milk samples with AFM_1_ levels below 5 ppt were considered negative.

#### Statistical analysis

The SPSS version 20 software was used for the statistical evaluation of the data. The data indicated by counting in the applied questionnaire form were evaluated as numbers and percentages. It was determined that various variables showed normal distribution according to Kolmogorov-Smirnov test in the differences between AFM_1_ levels in breast milk. In this distribution, the independent t-test was used for binary variables, and the ANOVA test was used for multiple variables. AFM_1_ levels, age, educational status, and nutritional characteristics of mothers were examined by linear regression method. The data were analyzed within the 95% confidence interval, and the significance level was taken as p<0.05.

## RESULTS

The mean age of the women participating in the study was 28±5.56. Educational status varies, where 27.8% are primary school graduates and 26.7% are university graduates. Notably, 30% of women had a spontaneous vaginal delivery, and 70% had a cesarean section. It was determined that 12.2% of them took iron medicine, and 6.7% took vitamin D as supplements during the lactation period. In addition, it was seen that 4.4% of them used the herbal mixture, mainly lemon water and milk tea, in this period (Table 1).

**Table 1.** Sociodemographic characteristics of mothers.

Variable	Value
Participants (N)	90 (100)
Age	
Mean±SD	28±5.56
Type of birth, n (%)	
Vaginal delivery	27 (30.0)
Caesarean delivery	63 (70)
Educational status, n (%)	
Primary education	25 (27.8)
Middle school	15 (16.7)
High school	19 (21.1)
College	24 (26.7)
Master’s-PhD	7 (7.8)
Living place, n (%)	
Village-town	10 (10.1)
District	32 (35.6)
City center	48 (53.3)
Working condition, n (%)	
Housewife	70 (77.8)
Public officer	8 (8.9)
Employee	8 (8.9)
Academician	4 (4.4)
Smoking, n (%)	
Yes	6 (6.7)
No	84 (93.3)
Chronic disease, n (%)	
Yes*	15 (16.7)
No	75 (83.3)
Using vitamins, n (%)	
Yes	17 (18.9)
No	73 (81.1)
Vitamins used, n (%)	
None	73 (81.1)
Vitamin D	6 (6.7)
Iron	11 (12.2)
Use of herbal mixture, n (%)	
Yes	4 (4.4)
No	86 (95.6)

*Thyroid (6.7%), hypertension (3.3%), diabetes (2.2%), asthma (2.2%).

In this study, 12.2% of the women defined their houses as damp and 2.2% as quite damp. It was determined that 54.4% of the women used ready-made dairy products, and 81.1% boiled them. It was determined that 81.1% of them consumed spices, and 10% of the spices they used in their homes were moldy. It was determined that 64.4% of women consumed dried fruits and vegetables. The rate of those who read on the packages in grocery shopping is 77.8%. It was determined that 71.1% of them read the expiration date, and 4.4% read the content. It was observed that 54.4% of the women participating in the study did not consume any acidic beverage products, 51.1% of them did not consume any ready-made food products, and 37.8% of them never consumed any processed food products.

The AFM_1_ levels in breast milk were found to be positive in 83.3% and negative in 16.7%. The mean AFM_1_ ratio in positive samples was measured as 12.16±5.85 pg/ml (5.00–23.18 pg/ml). The relationship between mothers’ AFM_1_ levels and age, education level, region of residence, and house dampness was analyzed by linear regression analysis. The regression model was statistically insignificant (F=1.099; p<0.373, Table 2).

**Table 2.** Regression analysis results for AFM_1_ levels and nutritional status of the last 24 h.

	β_0_	β_1_	SH.	Confidence interval (95%CI)	Test statistics	p	r_1_	r_2_
Constant	13.47	0	4.29	4.92, 22.01	3.13	0.00	0	0
Age	-0.00	-0.00	0.11	-0.24, 0.22	-0.07	0.94	-0.04	-0.00
Educational status (Primary education)								
Middle school	3.03	0.19	2.06	-1.06, 7.13	1.47	0.14	0.23	0.16
High school	1.10	0.07	1.83	-2.53, 4.74	0.60	0.54	0.09	0.06
College	-2.29	-0.17	1.73	-5.74, 1.15	-1.32	0.18	-0.25	-0.14
Master’s/PhD	-0.47	-0.02	2.56	-5.59, 4.63	-0.18	0.85	-0.04	-0.02
Living place (Village-town)								
District	-1.78	-0.14	2.19	-6.14, 2.57	-0.81	0.41	0.02	-0.09
City center	-1.79	-0.15	2.10	-5.91, 2.40	-0.85	0.39	-0.1	-0.09
Dampness at home (No damp)								
Damp	-0.00	0	1.94	-3.87, 3.86	-0.00	0.99	0.02	0
Quite damp	0.45	0.01	4.24	-8.00, 8.90	0.10	0.91	-0.02	0.01

β_0_: unstandardized coefficient; β_1_: standardized coefficient; r_1_: simple correlation; r_2_: partial correlation.

The relationship between mothers’ AFM_1_ levels and their consumption of ready-made, processed, and acidic foods were analyzed by linear regression analysis. The regression model was statistically insignificant (F=1.091; p<0.375). Although the model was insignificant, a relationship was found between the AFM_1_ level and the frequency of consuming processed food (p=0.04). It was observed that the AFM_1_ value decreased by 3.22 times when consuming processed products 1–2 times a month. It has been observed that consuming ready-made and acidic foods is not effective in the AFM_1_ level.

The relationship between the mothers’ AFM_1_ levels and their nutritional status in the last 24 h was analyzed by linear regression analysis, showing statistically insignificant (F=0.269; p<0.847, Table 3).

**Table 3.** Regression analysis results for AFM1 levels and nutritional status of the last 24 h.

	β_0_	β_1_	SH.	Confidence interval (95%CI)	Test statistics	p	r_1_	r_2_
Constant	14.64		2.45	9.77, 19.52	5.97	0.000		
Consuming dairy products in the last 24 ho (Yes)								
No	-0.03	-0.00	1.33	-2.68, 2.62	-0.02	0.98	0.01	-0.00
Consuming fruit in the last 24 h (Yes)								
No	-0.93	-0.08	1.42	-3.77, 1.89	-0.65	0.51	-0.08	-0.07
Consuming fat in the last 24 h (Yes)								
No	1.61	0.12	1.44	-1.26, 4.48	1.11	0.26	0.05	0.12
Consuming bread in the last 24 h (Yes)								
No	-0.10	-0.00	2.10	-4.28, 4.08	-0.04	0.96	-0.06	-0.00
Consuming vegetable in the last 24 h (Yes)								
No	-0.05	-0.00	1.32	-2.68, 2.56	-0.04	0.96	0.00	-0.00
Consuming meat in the last 24 h (Yes)								
No	-2.06	-0.15	1.50	-5.04, 0.92	-1.37	0.17	-0.13	-0.15
Consuming liquid food in the last 24 h (Yes)								
No	-2.35	-0.16	1.62	-5.59, 0.88	-1.44	0.15	-0.16	-0.15

β_0_: unstandardized coefficient; β_1_: standardized coefficient; r_1_: simple correlation; r_2_: partial correlation.

## DISCUSSION

In this study, AFM_1_ was detected in 83.3% of 90 breast milk samples. The literature review found that it is seen in 93.8% in Lebanon, 98.1% in Iran, 92 and 99.5% in the UAE, 10.5% in Turkey, 90% in Colombia, and 89% in Mexico^4,8,9,12,16,17,18,19,20,21^. AFM_1_ was found to be positive (≥5 pg/mL) in those aged 26–35years, primary school graduates, and living in the city center more than women with a mean age of 28±5.56 years. This situation is similar to the study conducted in Lebanon; it was lower than Iran, Mexico, and the UAE rates^9,12,17–19^.

Since mycotoxins are carcinogenic, mutagenic, teratogenic, and toxic substances, it is crucial to determine their levels^2^. Thus, many related studies have been carried out in Turkey. Cherkani-Hassani et al. scanned the studies between 1984 and 2015 to determine the prevalence, levels, and exposure conditions of mycotoxins and their metabolites in breast milk and found that 63 studies were conducted worldwide^22^. The majority of these studies were conducted in Egypt, Iran, Italy, Sudan, and Turkey; however, it is reported that the highest AFM_1_ values are observed in Egypt, Ghana, UAE, Nigeria, and Sierra Leone^22^. In a study examining the relationship between maternal dietary habits and mycotoxin in Italy, only 4 out of 82 milk samples were AFM_1_ positive. It was concluded that mycotoxin was significantly higher in the milk of those who consumed bread, bakery products, and processed pork^14^. In a study conducted in Egypt, 138 of 388 breast milk samples were AFM_1_ positive^20^. In a study conducted in Sudan, 51 of 94 breast milk samples were AFM_1_ positive. The main sources of this are peanut butter, vegetable oils, and rice^23^. In a study conducted in Jordan, all 80 breast milk samples were positive for AFM_1_; the average of AFM_1_ is 67.78 ng/kg. It has been determined that consumed grain products cause this contamination^24^. In a study conducted in China to determine the effects of seasonal differences on the AFM_1_ level of milk, 43 of 72 raw milk samples were found to be AFM_1_ positive. It was noted that the AFM_1_ concentration increased during the winter months, and seasonal variation was noted. Therefore, people who consume this raw milk are affected in the same way^25^. In a study conducted in Northern Iran, 39 of 250 breast milk samples were AFM_1_ positive. AFM_1_ levels in rural areas were higher than in the city center. It has been estimated to be due to dietary habits in rural areas that tend to consume more bread, rice, and non-alcoholic beer^26^. In another related study conducted in Morocco, 43 out of 82 breast milk samples were positive for AFM_1_^22,27^.

In the meta-analysis examining the prevalence and concentration of AFM_1_ in breast milk based on the socioeconomic indices and precipitation of mothers, it was reported that the prevalence of AFM_1_ in breast milk increased significantly with increasing annual mean precipitation and poverty^5^. This result is similar to our study. It was determined that the high levels of AFM_1_ in the mothers’ milk were due to the high annual precipitation rate of the region where the research was conducted.

Studies related to this case in Turkey cover the provinces of Afyonkarahisar^4^, Istanbul^28^, Ankara^2^, Erzurum^29^, Şanlıurfa^27^, Eskişehir^30^, and the district of Fethiye^3^. According to the results of these studies, the highest value in milk samples collected in these regions was determined in Ankara, with a range of 60.90–299.99 ng/L. In the study conducted in Afyonkarahisar, 10.5% of 200 breast milk samples were found positive; positive samples had a mean of 8.45 pg/mL AFM_1_^4^. In the study conducted in Istanbul to measure AFM_1_ levels in breast milk and raw milk, 8 out of 61 breast milk samples were found to be AFM_1_ positive; in positive samples, the mean score was 5.68 ng/L. In this study, no relationship was found between the sociodemographic characteristics of women and their AFM_1_ exposure, and this study concluded that dried fruits, nuts, spices, and tea are the primary sources of AFM_1_ in breast milk, contradicting the common assumption. In addition, it was concluded that the positive cases were due to the high consumption of carton milk in Istanbul^28^. In the study conducted in Ankara, AFM_1_ was positive in all 75 breast milk samples. In the study conducted to determine the relationship between moldy cheese consumed in Erzurum and AFM_1_ levels in breast milk samples, AFM_1_ was positive in 18 of 73 breast milk samples. AFM_1_ was positive in 12 of 44 women who consumed moldy cheese. However, they concluded that there was no difference between AFM_1_ exposure in those who consumed and did not consume moldy cheese^29^. In the study conducted in Sanliurfa, it was investigated whether there is a seasonal difference in AFM_1_ levels in breast milk. Of the 74 breast milk samples, 66 were positive for AFM_1_, and a mean of 19.0±13.0 ng/l AFM_1_ was found in positive samples. Notably, 91.2% of 34 breast milk samples taken in June and 87.5% of 40 breast milk samples taken in December were AFM_1_ positive. They concluded that the exposure was higher in June^27^. In the study in Eskisehir, in which breast milk AFM_1_ levels were evaluated along with their relationship with the mother’s nutritional habits, only three of the samples were positive for AFM_1_ (mean; 3.59 ng/L). No relationship was found between mothers’ dietary habits and AFM_1_ level^30^. In the study conducted in Fethiye, 53 of 100 breast milk samples were positive for AFM_1_, and the mean of positive cases was 6.36 ng/L AFM_1_. A significant difference (58.8%) was found in the breast milk samples of homemakers compared to the workers^3^. In the present study, while the educational status of women was associated with AFM_1_ levels, their place of residence and age were not associated with AFM_1_ levels. Unlike all other studies, the AFM_1_ level was higher in women who consumed ready-to-eat food than those who did not. Whatever the reason, the high prevalence (83.3%) of AFM_1_ found in human milk in the present study shows the need to assess further the extent of exposure to aflatoxins in Turkey and the impact that this has on public health.

In the literature, it has been observed that the foods that affect the AFM_1_ level in breast milk include contaminated corn oil, peanuts, raw milk, peanut butter, vegetable oil, rice, cow’s milk consumption, soybean, and wheat flour^5^. In this study, however, no relationship was found between the consumption of ready-made milk and dairy products and acidic food consumption, and the level of AFM_1_. On the contrary, consumption of processed food (sausage) was associated with AFM_1_ level. This result in our study added a new product to the food contaminated with AFM_1_ level.

## CONCLUSIONS

The AFM_1_ result in this study was found to be higher than the studies conducted in Turkey. The reason for this is thought to be due to the heavy rainfall in the region where the study was conducted. It has also been observed that mothers are effective in consuming processed food. To minimize the possible harms of aflatoxin to women and newborns, safe food, storage conditions, and possible harm should be mentioned in pre- and post-pregnancy education.

## ETHICAL APPROVAL

Permission was obtained from the Human Research Ethics Committee of the Karabuk University (dated May 30, 2018, and no. 6/15) and the institute where the research was conducted. The study was based in accordance with the Declaration of Helsinki.

## AVAILABILITY OF DATA AND MATERIALS

All data are publicly available.
